# Viability of Web-Based Respondent-Driven Sampling of Belgian Men Who Have Sex With Men: Process Evaluation

**DOI:** 10.2196/60884

**Published:** 2025-05-05

**Authors:** Estrelle Thunnissen, Veerle Buffel, Linda Campbell, Bea Vuylsteke, Philippe Bos, Edwin Wouters

**Affiliations:** 1 Centre for Population, Family and Health Department of Sociology University of Antwerp Antwerp Belgium; 2 Brussels Institute for Social and Population Studies Vrije Universiteit Brussel Brussel Belgium; 3 Department of Public Health Institute of Tropical Medicine Antwerp Belgium

**Keywords:** web-based respondent-driven sampling, men who have sex with men, Medical Resource Council framework, population inference, nonparticipation, overresearch, survey fatigue

## Abstract

**Background:**

Obtaining a representative sample is a substantial challenge when undertaking health research among hidden and hard-to-reach populations such as men who have sex with men Web-based respondent-driven sampling (WEB RDS) was developed to overcome such sampling challenges and to create population estimates based on network and sampling characteristics. Despite a decade of research, it remains unclear whether WEB RDS is suitable for sampling hidden populations such as men who have sex with men.

**Objective:**

This study aims to evaluate how viable the WEB RDS methodology is for obtaining a nationwide sample of men who have sex with men, suitable for population inference of sexual health characteristics, in Belgium.

**Methods:**

We adapted the Medical Resource Council process evaluation framework for interventions, to evaluate an empirical WEB RDS. Viewing “WEB RDS” as a complex intervention with respondent-driven recruitment as the aim, we evaluated indicators of context, implementation, mechanisms of impact, and performance. We analyzed the data using a mixed methods approach that integrated findings from quantitative analysis, such as RDS diagnostics, and qualitative thematic analysis.

**Results:**

Sampling did not reach a sufficient sample size (n=193) to compensate for an RDS design effect of 3 and the number of recruitment waves was low (waves=7). A visual examination of the convergence and bottleneck plots indicates that many more waves of recruitment would be needed for population estimates to become independent of the seeds. However, producing further waves was impeded by challenges inherent to the research context and process. Men who have sex with men and their community organization representatives indicated that, in Belgium, men who have sex with men are overresearched, with low motivation for the topic of sexual health and digital etiquette dictating not sharing survey links. A moderate reward of €10-€30 (US $11.2-$33.6) with a dual incentive structure was insufficient to overcome these barriers.

**Conclusions:**

This study indicates that WEB RDS, even with a moderate incentive, is not a viable sampling strategy for obtaining valid population estimates of sexual health traits of men who have sex with men in Belgium. The study emphasizes the need to understand men who have sex with men research motivation and topic saliency. Additionally, the study highlights the importance of digital etiquette. Finally, the study showcases the use of the adapted Medical Research Council framework for evaluating WEB RDS methodology.

## Introduction

### Sampling Challenges With Men Who Have Sex With Men

The challenges of conducting health research among hard-to-reach groups, such as men who have sex with men, are well-known. These challenges are particularly evident in obtaining a representative sample from populations without a sampling frame [1,2]. This is the case in Belgium (Flanders) where representative population-based data on sexual orientation is not included in current large and representative population surveys (eg, Belgium health interview survey) or register data (eg, health insurance data) [3]. There is evidence that numerous methods for sampling men who have sex with men, including venue-based sampling, web-based sampling, or sampling through sexual networking applications, may be inherently biased and may therefore create a distorted image of the population, missing important subpopulations such as closeted gay men, bisexuals, immigrants, and those not participating in the gay scene [4-10]. Therefore, there is a pressing need to develop and use sampling methods that aim to obtain samples of men who have sex with men and other hard-to-reach groups that can be used for population inference. Population inference is crucial for interpreting previous research findings and supporting service planning.

### Respondent-Driven Sampling for Overcoming Sampling Challenges

Respondent-driven sampling (RDS) was developed to overcome sampling challenges in studies of populations (such as men who have sex with men) where a sampling frame is difficult to define [11]. RDS uses chain referrals (respondent-to-respondent invitations) with structured incentives (payment at set participation goals) as a recruitment strategy. Subsequently, the social network parameters of participants are used to weigh the data, resulting in estimates of population characteristics [12]. However, web-based respondent-driven sampling (WEB RDS) as a sampling method requires that certain assumptions are met (eg, with-replacement sampling, connected networks, random recruitment, seeds with probability proportional to degree) [13,14] and valid estimates can be generated via weighing procedures only under certain estimator specific conditions. It remains unclear how often these conditions are met, as many studies do not report the necessary information [15,16]. Despite these limitations, RDS methods are considered to be a promising avenue for sampling men who have sex with men and creating population estimates of this invisible population.

### Methodological Innovation: WEB RDS

In the early 2010s, a new variant of RDS emerged—WEB RDS—where respondents recruit contacts through digital invitations. Unlike traditional RDS, WEB RDS is only suitable for countries with high levels of smartphone or computer ownership and good internet coverage. In these contexts, WEB RDS may actually be preferred to standard RDS practices, as it offers participants anonymity and ease of participation [17,18]. Additionally, unlike standard RDS practices, WEB RDS is commonly used among nonmarginalized populations, such as the general population and students [19]. Access to a larger geographical area and larger population are considered major benefits of WEB RDS compared to RDS. In the past decade, several studies have reported successful WEB RDS implementation [17,20-24] and this method is currently considered promising for sampling LGBTQI+ (lesbian, gay, bisexual, transgender, queer/questioning, intersex, and others) populations [25].

### Gap: Viability of WEB RDS

Nonetheless, despite a decade of research, it remains undecided whether WEB RDS is suitable for sampling men who have sex with men with the aim of population inference [26]. Underreporting of sample diagnostics and formative research in the literature [19] means that it is unclear whether sampling was indeed successful in many previous studies. A scoping review of WEB RDS found that only 11% of studies assessed for eligibility provided adequate recruitment details and of those only 28% were suitable for population inference. Among these were 3 WEB RDS studies involving men who have sex with men. Two studies set in Vietnam reached equilibrium and were therefore suitable for population inference [17,18], while another study set in Sweden had short recruitment chains and did not reach equilibrium [26]. The difference in the length of recruitment chains between the studies in Vietnam and Sweden may be due to different contexts. Vietnam is a lower- to middle-income country while Sweden is a High-income country (HIC), and this difference may impact the effect of the incentive. Furthermore, same-sex relationships face greater stigma in Vietnam compared to Sweden, where they are more widely accepted although some stigma still remains [27,28]. In countries where acceptance of sexual minorities is low, protection of LGBTQI+ rights is insufficient, and sexual health services are hard to access, the population may be more motivated to engage in research aimed at improving their circumstances. WEB RDS studies that rely primarily on internal motivation, combined with a low-to-moderate incentive, may not be suitable for men who have sex with men populations in high-income settings with relatively lower levels of stigma; motivation may be low due to relatively better rights protection and sexual health services access [26-28]. To the best of our knowledge, to date, no further evidence has emerged to assess the suitability of WEB RDS for population inference of men who have sex with men characteristics in high-income settings.

This study aims to evaluate how viable the WEB RDS methodology is for obtaining a nationwide sample of men who have sex with men, suitable for population inference of sexual health characteristics, in Belgium. We rely on the analytical framework of Helms et al [19]—which itself is an adaptation of the Medical Resource Council (MRC) process evaluation framework [29]—that views sampling as an intervention with the aim of recruiting people into the study.

## Methods

### Data Collection

#### WEB RDS Design

We developed and conducted a pilot study, followed by the main study. The main WEB RDS was initiated on February 28, 2022, and the last participant was introduced on March 28, 2022. The survey closed on April 30, 2022, two weeks after the last questionnaire was completed. We recruited an initial pool of participants (seeds) who were able to invite up to 6 others digitally, who were then able to participate and in turn recruit up to 6 others, ad infinitum. While no incentives were provided during the pilot, during the main study participants received €10 (the conversion rate at the time of the study was €1 to US $1.12) for participation and €5 for each successfully recruited contact [26,30]. Additionally, we halved the survey length from approximately 20 minutes during the pilot to approximately 10 minutes during the main study. Multimedia Appendix 1 presents details of the 2 sampling designs, following the Strengthening the Reporting of Observational Studies in Epidemiology for Respondent-Driven Sampling (STROBE-RDS) report checklist for RDS study design [31].

#### Quantitative Data

For the process evaluation, quantitative data such as survey and recruitment data from the pilot and the main WEB RDS study were collected through “National Institute for Public Health and the Environment RDS” software [32]. The software assigns unique identifiers to respondents and uses this to track the recruitment process. The recruitment data consisted of identifier of the “parent” (recruiter of respondent), identifiers of the “children” (recruited by the respondent), number of “children,” and status of the invitations sent (no response, invitation accepted but survey not completed, and survey completed). Respondent network size was measured by asking: “How many Men who have Sex with Men do you know (have contact information of)?” This information was used to compile an RDS data frame. Additional survey data included variables related to socioeconomic status, sexual health, and sexual behavior.

The process evaluation used 4 variables which were all traits suspected to be biased in convenience samples of men who have sex with men, namely age, education, urban residence, and having only anonymous partners. The last variable was included as based on a hypothesis from our formative research; that men who only have anonymous sex might not be reached through WEB RDS. For this trait a proxy was used; only having sex with occasional partners. All variables were categorized into binary variables to aid the graphic analysis, as piloted and described by Gile et al [33].

#### Qualitative Data

For the process evaluation, qualitative data were collected as follows: (1) a literature review was performed on WEB RDS recruitment mechanisms related to HICs and the men who have sex with men population; (2) community expert interviews; (3) RDS expert interviews; (4) focus group discussions with community stakeholders were held to better understand the nationwide network structure of Belgian men who have sex with men, the value of incentives optimal for participation and recruitment, and potential barriers and facilitators to recruitment and participation; (5) a pilot study was subsequently performed among the target population; (6) after the pilot, seeds who had not participated or recruited were contacted for a short nonresponse interview (maximum 10 minutes) by phone asking for information on why that was the case. In case individuals recruited by the seed had not participated, seeds were asked why they thought their recruits had not participated; (7) RDS expert consultations and (8) community stakeholder consultations were held to interpret the pilot results and nonresponse interviews, and to develop an incentive structure to be implemented in the study; after the WEB RDS study, (9) further RDS expert consultations and (10) community stakeholder consultations were held to interpret the results. Furthermore, research documents, logbooks, and correspondence between researchers were collected for analysis. For more details on the amount and type of qualitative data collected, see Multimedia Appendix 2.

### Analysis

#### MRC Process Evaluation Framework

**Figure 1:**
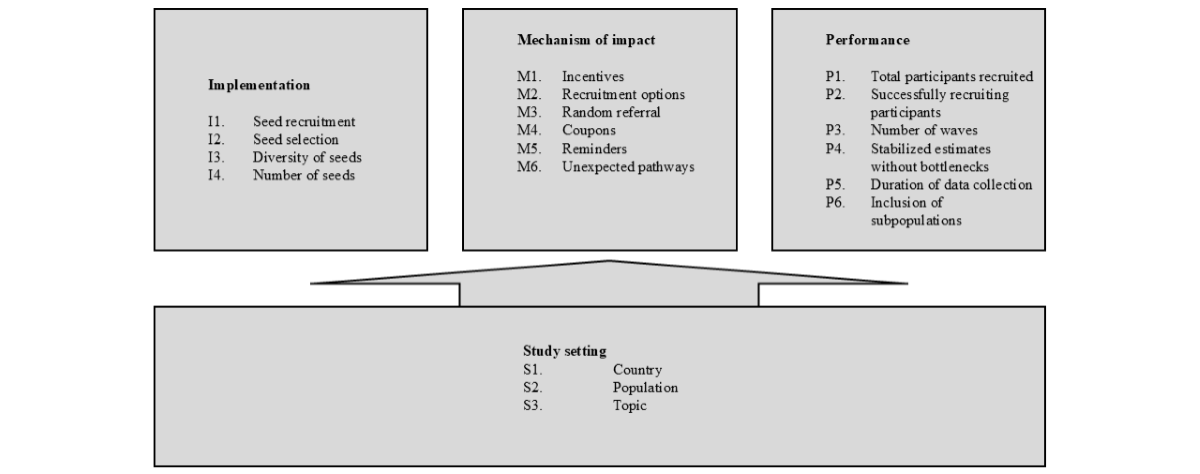
Web-based respondent-driven sampling evaluation framework.

We used and expanded on the analytical framework developed by Helms et al [19], which itself was adapted from the MRC process evaluation framework [29] to evaluate WEB RDS studies for their systematic review. We made appropriate modifications for evaluating an empirical WEB RDS study (Figure 1) based on the MRC process evaluation framework [29]. We implemented sampling by recruiting men who have sex with men and asking them to invite peers, with the aim that respondents subsequently recruit others into the sample through various mechanisms of impact, such as incentives for participation and recruitment. See Figure 1, adapted from Helms et al [19], for the indicators of implementation, mechanisms of impact, performance, and study setting. A short explanation of each indicator is provided in Multimedia Appendix 3.

To evaluate the indicators listed (Figure 1 and Multimedia Appendix 3), we performed and combined quantitative and qualitative analysis. Survey and recruitment data were analyzed using the *RDS* package in R (R Foundation for Statistical Computing) and IBM SPSS, adhering to the RDS diagnostics approach developed by Gile et al [33]. Qualitative data were analyzed and codes were generated using a manual thematic inductive approach [34,35]. Themes were categorized into broader descriptive categories, and findings from the quantitative analysis were integrated into the themes at this phase. A coherent line of argument was subsequently generated.

#### Study Setting

Qualitative data on the indicators of context, namely a HIC with relatively low levels of stigma and good LGBTQI+ rights protection and sexual health services (S1), population network structure (S2), and the sexual health topic (S3), were coded and themes generated. Qualitative data on population network structure (S2) was compared to recruitment homophily (similarity of recruiter and recruited on traits of interest); however, a valid population homophily could not be estimated or evaluated due to inadequate performance. The country (S1) and sexual health topic (S3) indicators were combined with quantitative and qualitative data on incentives (M1), unexpected pathways (M6), and performance (P1-P4, P6).

#### Implementation

Implementation was analyzed through social and geographical mapping of the seeds, which consisted of reporting on and analyzing the number of seeds (I4) and their diversity of traits of interest (I3). Qualitative analysis was performed on the recruitment (I1) and selection (I2) process using research notes and a logbook. Synthesis was reached by interpreting the seed mapping results in light of the thematic analysis of our recruitment and selection processes.

#### Mechanisms of Impact

Qualitative data related to incentives (M1), random referral (M3), and unexpected pathways (M6) were coded and themes were generated. Furthermore, the effectiveness of incentives (M1) was analyzed by comparing the performance (P1, P3) of the pilot (without material incentives, length 20 minutes) to the main WEB RDS study (with material incentives, length 10 minutes). These findings were then integrated into the thematic analysis. There were insufficient data for qualitative or quantitative analysis of recruitment options (M2), coupons (M4), and reminders (M5).

#### Performance

First, we evaluated whether the total number of participants recruited (P1) was sufficient to compensate for the RDS design effect of 3 with an SE of 0.03, as most recently recommended for RDS [36,37]. We calculated the minimum sample size (Multimedia Appendix 4) using the formulae outlined by Salganik [37], and this resulted in a target sample size of 625 participants. The calculation can be found in Multimedia Appendix 4. Second, we examined participants who were recruited successfully (P2) through participation and recruitment rates. Third, we compared the number of waves (P3) to standards for sufficient waves in the field. Fourth, the dynamics of the RDS estimates were explored using a graphical approach to determine if the estimates had stabilized without bottlenecks (P4) [33]. This was carried out by a visual examination of the convergence plots (estimates over time for the total sample), bottleneck plots (estimates over time per chain), and all-points plots (inclusion of traits over time), as suggested by Gile et al [33]. The estimator used was RDS-II, also called the Volz-Heckathorn estimator [13].

Finally, to evaluate the inclusion of subpopulations (P6) of interest, we compared sociodemographics of the WEB RDS sample to sociodemographics of a cross-sectional convenience sample collected on the web from the European Men Who Have Sex With Men Internet Survey (EMIS) [38]. Data on the duration of the data collection indicator (P5) were not informative and hence were not analyzed in-depth.

### Ethical Considerations

The study was approved by the ethical committee for the Social Sciences and Humanities of the University of Antwerp (SHW_17_64). All participants provided written informed consent in their chosen language to participate. The dataset will not be published, as the dataset contains sensitive information on gender identity and sexual behavior, and participants did not provide explicit informed consent for data to be published. Aggregate data can be shared upon reasonable request. Contact information provided by seeds and respondents who wanted to receive the incentives was decoupled from their survey results automatically by the RDS software and data was de-identified. Participants were compensated with €10 for filling out the survey and €5 per successfully recruited participant up to a total amount of €30.

## Results

### Study Setting

RDS experts expected that incentivizing a target population in a high-income setting (S1) would be more difficult than in a low-income setting (S1) impacting how incentive (M1) functions. Further results on incentives are presented under the Mechanisms of Impact section.

**Figure 2:**
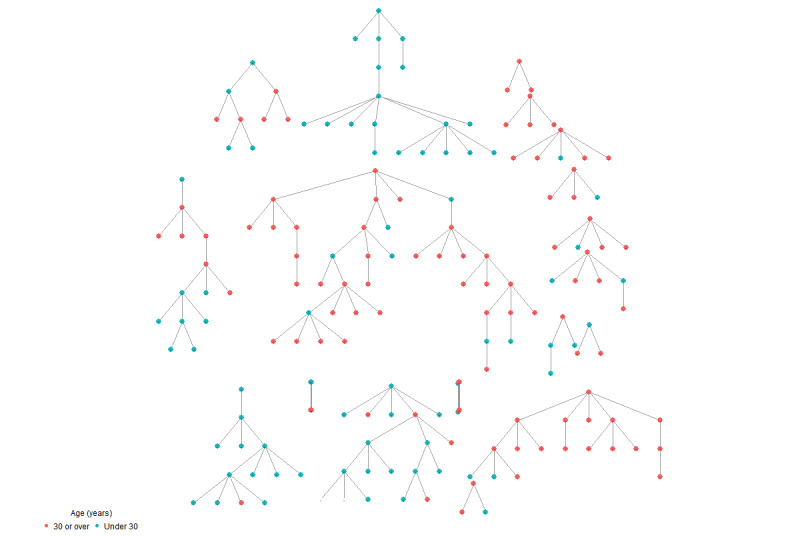
All-point plot of age.

**Figure 3:**
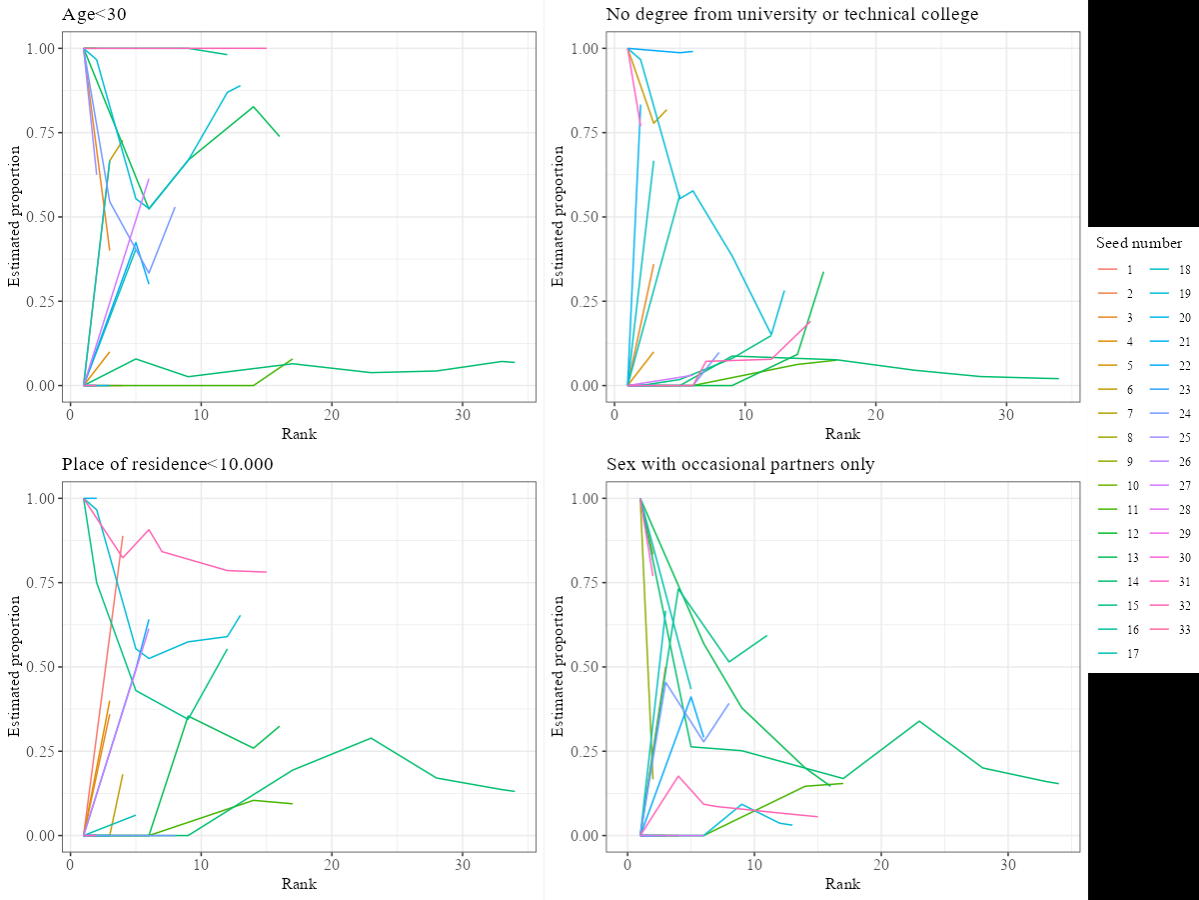
Bottleneck plot.

Community representatives had doubts about whether Flemish men who have sex with men (S2) could be considered as one single network because men who have sex with men networks are likely to be localized at the city or county level; however, our sample data indicated only modest recruitment homophily on the living environment (homophily value=1.10). While clustering on age was not mentioned in interviews or focus groups, our analysis showed that the highest degree of recruitment homophily was associated with age (recruitment homophily=1.43). Both the all-points plot (Figure 2) and bottleneck plot (Figure 3) illustrate that the trait is highly chain-dependent. The all-points plot specifically demonstrates a marked increase in people under thirty years of age entering the sample late and predominantly in one chain (the longest). The all-point plots only include seeds who participated and successfully recruited others (N=23) while the bottleneck plots only include seeds who participated and gave their network size (N=33). For all-point plots of the other traits, see Multimedia Appendices 5-7.

Community representatives also expected that the subpopulation (P6) of Belgian men who have sex with men (S2) who do not identify as gay would not be part of any men who have sex with men network since they often participate anonymously in the men who have sex with men milieu. Nonetheless, we found that 25 people who only had sex with occasional partners were recruited by their peers. Additionally, there was no significant correlation between only having sex with occasional partners and network size or successful recruitment of contacts. There was no recruitment homophily (1.02) on this trait.

We found evidence that the topic (S3) was an unexpected pathway (M6), specifically acting as a barrier to both participation and recruitment. These results are shown under the Mechanisms of Impact section.

**Figure 4:**
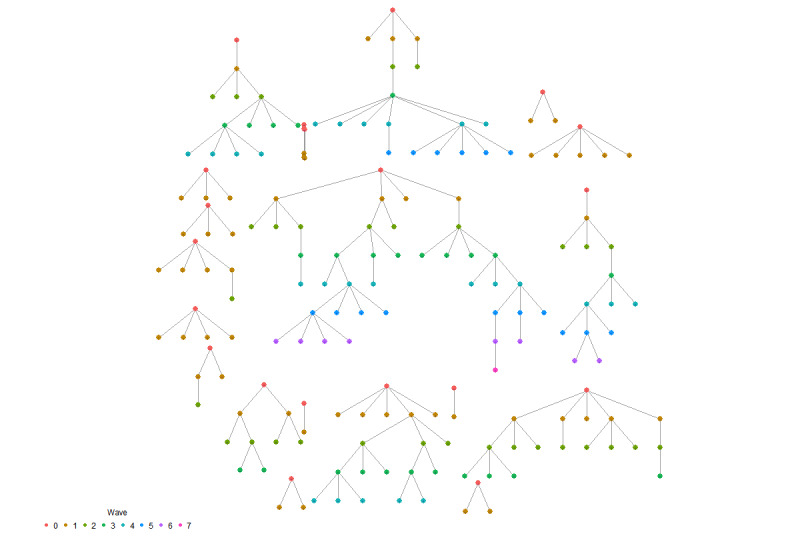
Recruitment tree.

### Implementation

Previous convenience sampling among men who have sex with men in Belgium has shown that there is often an underrepresentation of those with lower levels of education, those residing in rural areas, and those younger than 30 years of age. We, therefore, actively pursued potential seeds with these characteristics during seed recruitment (I1). Furthermore, as formative research cast doubts on whether Belgian men who have sex with men form a single national network, we strove to include geographically dispersed seeds (I1). However, since the majority of seeds were recruited from the researchers’ networks (I1), the diversity of seeds (I3) was constrained, with an overrepresentation of seeds with college or university degrees from Brussels, Antwerp, and Gent (East-Flanders) [39]. More information on seed characteristics is provided in Multimedia Appendix 8. Selection of seeds (I2) based on diversity and motivation from our seed pool was impractical. The limited number of potential seeds recruited (I4) and the shortness of recruitment chains (P3) meant that we had to use all seeds collected (n=38) to try to attain the target sample size (P1). Not selecting on motivation may have led to a high percentage of seeds who did not participate (n=4, 11%) or who did participate but did not invite contacts (n=13, 38%). After adding the last seed on March 28, 2022, the survey petered out on its own. As adding new seeds hadn’t been fruitful, and would likely result in more short chains instead of chains long enough for valid population estimation, we did not restart seed recruitment.

### Mechanisms of Impact

#### Incentives

Thematic analysis of the indicators of mechanisms of impact resulted in rich data on incentives (M1), random referral (M2), and unexpected pathways (M6). Both RDS and community experts advised us to provide material incentives (M1). Community experts framed the need for incentives to show appreciation for the target population, create goodwill, show that their participation is not taken for granted, and compensate people for their valuable time. Both community and RDS experts advised against a lottery ticket or a very small sum because they expected that would be perceived as insulting and underappreciative. The monetary incentive structure and amount (double incentive system, value ranging €10-€30 as an electronic gift card) were based on literature [26,30] and positive feedback from the community representatives. We investigated the impact of monetary incentives by comparing results from the pilot (without monetary incentives; 20 minutes) to the main WED RDS study (with monetary incentives; 10 minutes). The main study clearly outperformed the pilot: the pilot started with 24 seeds and ended spontaneously with 25 completed surveys after 3 waves. The main study began with 38 seeds and ended spontaneously with 151 completed surveys after 7 waves. We suspect the higher performance is due to the incentive provided. Although the survey length of the main study was half that of the pilot (10 minutes compared to 20 minutes), qualitative research did not indicate that this made an impact. Survey length was not mentioned as a barrier in the nonresponse interviews and there was little dropout while filling out the survey during the pilot. It is possible that the survey length had an invisible effect on the willingness to recruit; however, this could not be investigated further with the data available.

#### Random Referral

During consultations and project meetings, RDS experts advised us to forego randomization of recruitment (M2), despite it being an RDS assumption. They stated that randomization has proven too much of an obstacle for respondents and that random selection of contacts from a list is not very realistic, because people are reluctant to send invites to random contacts. Consequently, too many branches die off prematurely (eg, there is an insufficient number of waves [P3]). Community experts confirmed that randomly selecting a recruit from a list of (pseudonym) names was an unrealistic expectation. Not requiring random recruitment (M2) likely led to recruitment bias, and providing incentives (M1) may have exacerbated this bias. RDS experts stated that people sent invitations to those they believe will participate, to increase the chance of getting paid. They hypothesized that well-known activistic men who have sex with men with a large network are perceived as more likely to participate, and therefore, would be oversampled. We had insufficient quantitative data to confirm or deny this hypothesis.

#### Unexpected Pathways

Two unexpected pathways (M6) were identified: (1) survey fatigue, in the sense of being overresearched as a population in general (S2) and on the topic of sexual health (S3) in particular, and (2) digital etiquette discouraging sharing survey links. Community experts stated that men who have sex with men contacted by their organizations experienced general survey fatigue. The issue of survey fatigue and the overwhelming number of survey invitations encountered by the population were confirmed during nonresponse interviews after the pilot.

In addition to a general unwillingness to participate in surveys, topic-specific (S3) unwillingness was also reported. Community experts underlined that survey fatigue was especially high in sexual health surveys. They highlighted that gay men resent the unwarranted focus on their sexual practices, rather than on other aspects of their lives. During non-response phone interviews, respondents confirmed no motivation for the topic as one of the reasons for nonresponse (P2) among their contacts. The survey topic (S3) was also considered a barrier to recruiting others (P2); during non-response phone interviews after the pilot, men who have sex with men stated that they had not invited others due to the perceived complications and sensitive nature of sharing a survey on sexual health.

RDS experts also anticipated a cultural resistance to sharing survey links; digital etiquette discouraging spamming contacts in general and with survey requests in particular. Community organization representatives also expected digital etiquette to be a barrier to recruitment (P2) among Belgian men who have sex with men. They advised providing incentives (M1), as this could change the invitation from “spam” into “opportunity” and make people more comfortable inviting others to a web-based survey.

### Performance

**Figure 5:**
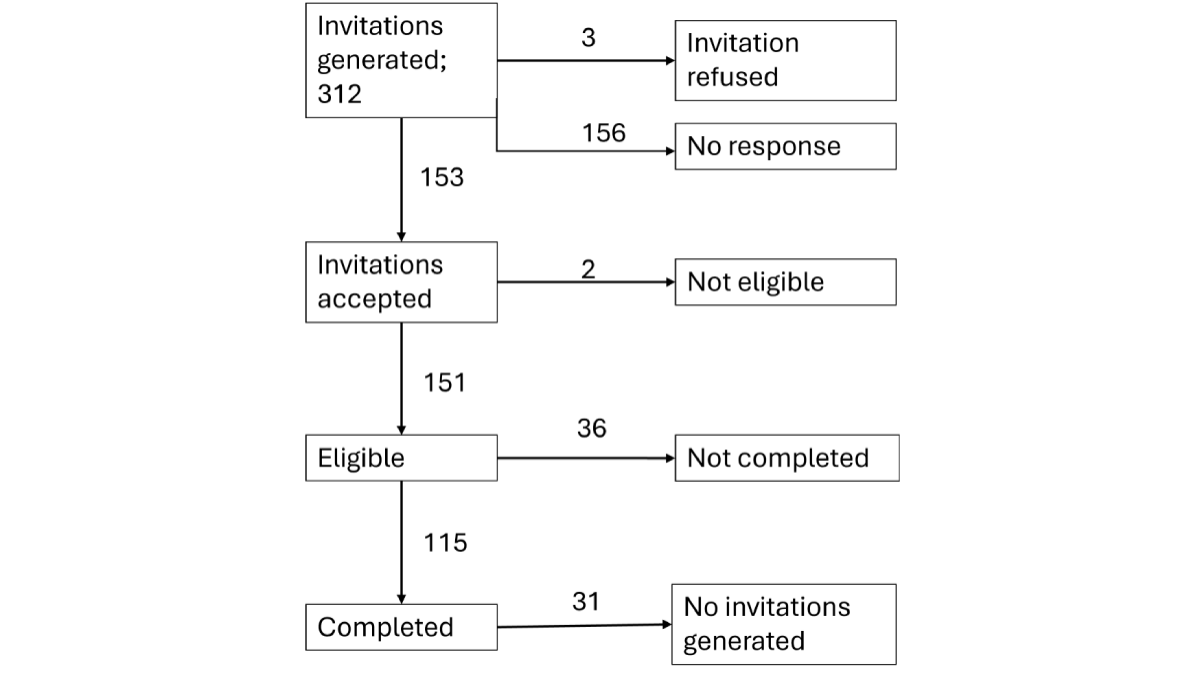
Respondent drop-out.

The sample size (P1; n=193) did not reach the target sample size of 625, which was necessary to compensate for an RDS design effect of 3. The number of waves (P3) was 7, which can be considered a short recruitment chain (Figure 4) [33]. The recruitment tree (Figure 4) only includes seeds who participated and recruited others (N=23). Regarding the success of participation and recruitment (P2), sampling died out predominantly due to nonresponse; more than half of the invitation links generated did not receive a response (Figure 5). “No links generated” was a further issue, with just over a fourth of respondents not generating links to invite contacts to the survey.

**Figure 6:**
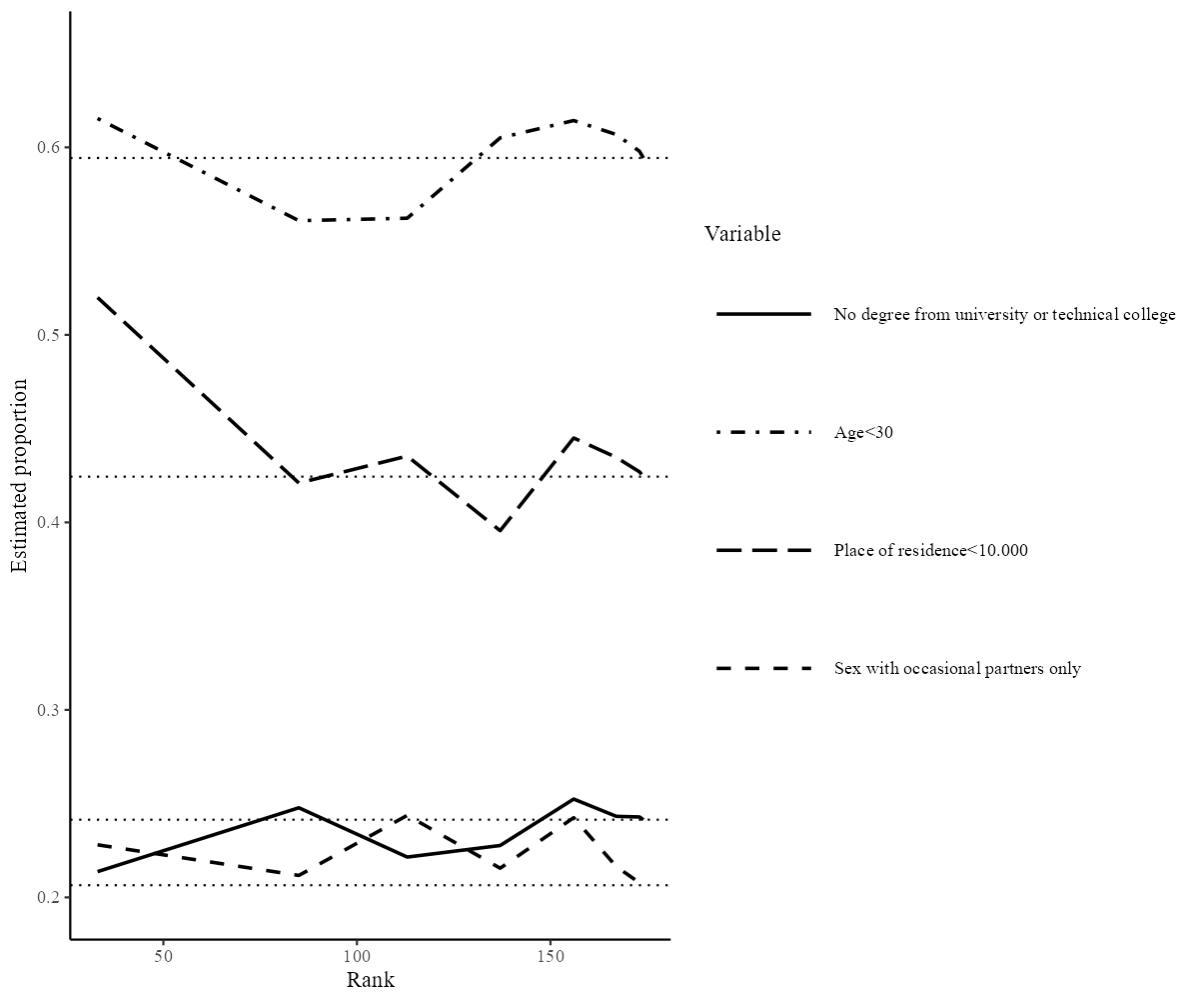
Convergence plot.

Regarding stabilization of the estimates without bottlenecks (P4), a visual examination of the changes in the population estimates as sampling progresses (as shown in the convergence plots, [Figure 6] and bottleneck plots [Figure 4]) indicates that the estimates do not converge on 1 value; the variability of the estimates remains quite large. This shows that the estimates have not yet stabilized and that they are still highly chain- and seed-dependent.

Regarding the inclusion of subpopulations (P6), we observed that the WEB RDS sample had a higher percentage of men who have sex with men under the age of 30 years (n=183, 42.1%) compared to the EMIS convenience sample in Belgium (n=492, 21.9%). This is remarkable since the sampling started with an initial overrepresentation of seeds aged 30 years and older and there was moderate recruitment homophily on age. Furthermore, the WEB RDS had a higher percentage of men who have sex with men from urban areas than the EMIS sample (n=136, 75.1% and n=1280, 57.5%, respectively) consistent with seed bias and modest recruitment bias toward including urban men who have sex with men in the sample.

In the WEB RDS sample, we found that men who have sex with men without a university or technical college degree were in the minority (n=40, 22%), aligning with a bias in the seeds and modest homophily on education (1.10). Additionally, 19% of participants (n=35) were men who have sex with men with only occasional partners, serving as our proxy for anonymous participation. Since EMIS measures different categories of sex partners, and years of education instead of degree attained, we were unable to compare these 2 variables. Multimedia Appendix 9 provides a complete overview of the inclusion of men who have sex with men with comparable traits in both samples, including data from complete and incomplete WEB RDS surveys.

## Discussion

### Summary

This study’s objective was to evaluate how viable WEB RDS methodology is for obtaining a sample of Belgian men who have sex with men suitable for population inference of sexual health characteristics. Through process evaluation, we found evidence that obtaining a WEB RDS sample suitable for population inference is not viable in the given research context. Diagnostics indicate that the final sample is not suitable for population inference and that many more waves would be needed for estimates to stabilize. However, in-depth analysis of quantitative and qualitative data revealed obstacles to producing more waves inherent in the research context. These include general and topic-specific survey fatigue among men who have sex with men, the topic of sexual health as a barrier to peer recruitment, and a digital etiquette of not sending surveys to contacts. While an incentive structure of €10-€30 substantially increased the sample size and number of waves compared to no incentive, with an indication that a moderate reward is perceived as respectful and appreciative, it was insufficient to overcome barriers to participation and recruitment.

### Limitations

There were several limitations to our process evaluation. First and foremost, while we followed the MRC process evaluation guidelines (developed for interventions) as well as the framework by Helms et al [19] as closely as possible, the fact that the method was not optimized to evaluate sampling may have led to missed opportunities regarding the richness of the results.

Second, both incentive structure and survey length were changed simultaneously, and while survey length was not ascertained as a barrier, we could not unequivocally conclude that adding incentives was the only reason for the improved response rate.

Third, our analysis of the men who have sex with men network structure was limited by the fact that the survey did not include location data (eg, questions on post code, address, municipality, province) and that valid population homophily could not be estimated.

Fourth, our nonparticipation analysis was limited; we could not contact individuals (who were not seeds) to inquire about their reasons for not participating in the study, as we neither had their contact information nor the informed consent to do so.

Fifth, the comparison with the convenience sample was limited since the significance of the difference in sample composition could not be determined.

Sixth, the proxy for men who have sex with men who participate anonymously in the men who have sex with men milieu and are “closeted” was not optimal; men who have sex with men who only had sex with occasional partners. This can include men who have sex with men who do share contact details with others and men who have sex with men who are “out.” Future WEB-RDS studies should include questions on concealment to assess whether “closeted” men who have sex with men have been reached [28].

Despite these limitations, this study makes a contribution to the literature that has been highly recommended in systematic reviews and methodological literature on (WEB) RDS and semiprobabilistic sampling of LGBTQI+: an in-depth analysis of the (WEB) RDS process among men who have sex with men [4,15,16,19,40].

### Comparison With Prior Work

#### Overresearch and Survey Fatigue

Our results on short recruitment chains align with those of Strömdahl et al [26], which suggests that WEB RDS with the goal of producing population estimates may not be viable among men who have sex with men in HICs, as long recruitment chains are necessary for valid inference [26]. Similar to Strömdahl et al [26], our study faced significant barriers to long recruitment chains, mainly nonresponse, likely linked to extreme survey fatigue among our target population. Further research into the extent and mechanisms behind survey fatigue among men who have sex with men is needed, not only for studies using WEB RDS but for other survey methods as well. Survey fatigue may be in line with a wider societal trend of web-based survey oversaturation and declining response rates [41-43]. In light of this, future studies could increase the number of coupons (more than 6) to compensate for the low survey participation rate and increase the sample size and length of recruitment chains. Survey fatigue may also be due to overresearch, where a particular population attracts disproportionate attention [44]. The latter could be an artifact of men who have sex with men being designated an at-risk population. The mechanism could be similar to spatial bias, where certain areas are more prone to being researched because of particular features of their populations, which in turn creates resistance to research within those populations [45]. If men who have sex with men–specific research fatigue exists, coordinating research among men who have sex with men within and among universities, governments, and community organizations could address this and would depend on political and social alignment between these organizations regarding research goals and aims.

Furthermore, topic-specific research fatigue among men who have sex with men is an additional concern for future studies. In our study, community organization representatives reported that their members were unhappy with the sheer number of studies conducted on their sexual behavior, versus other important aspects of their lives, and were thus unlikely to participate in studies on sexual health. The perception that researchers are driven by different agendas for their research populations can produce frustration with high levels of research attention and can lead to high levels of nonparticipation [46,47]. A study on nonresponse bias in surveys on HIV and sexuality found that topic salience plays a large role, with 20% declining to participate due to a lack of interest in the topic [48].

No motivation for the topic could also be a crucial barrier to respondent-driven recruitment; as men who have sex with men in a study by Diexer et al [30] indicated that motivation for the topic was the most important consideration for inviting contacts to a WEB RDS [30]. This motivation was considered far more important than a sufficient reward for inviting others. A systematic review of response rates to web-based surveys among men who have sex with men on various topics may shed further light on the research needs of men who have sex with men and their motivation for specific topics, without further overburdening the population. A preparatory, qualitative study into the perception among men who have sex with men about their research needs versus research encountered, their research participation history, and their preferred recruitment and data-sharing methods could also pave the way for future successful sampling.

#### Incentive Structure

The value of the incentive to be provided in a WEB RDS is a delicate balance. We found that €10 for participation and €5 per successful recruitment, up to a total of €30, was insufficient to overcome barriers among Belgian men who have sex with men. On the other hand, Wejnert et al [18] found that total compensation of US $55 was too high among American students, as it was perceived as a potential scam [18]. A similar study by Bauermeister et al [20] among American youths found that US $70 was successful for recruitment compared to US $30, which was not [20]. A study in Thailand showed that a WEB RDS could be performed there without incentives because contributing to research is culturally considered to not need a reward [49]. The need for a material incentive and a reasonable amount are likely country-, culture-, and population-specific. The fact that our main WEB RDS (with incentives) performed better than our pilot (without incentives) indicates that some form of material incentive is crucial for the sampling mechanism to work among men who have sex with men in Belgium. It remains to be seen, however, if increasing the amount would lead to more successful recruitment, as there are limits to what a conditional incentive can achieve. Marginal gains in participation decrease as the incentive gets higher—a participation plateau will be reached even when further increasing the incentive [50]. Our formative research indicates that a “reasonable” incentive functions as a sign of respect from researchers towards men who have sex with men; it shows that their participation is not taken for granted, which may be crucial when sampling populations with high levels of survey fatigue.

#### Digital Etiquette

During consultations and project meetings with WEB RDS researchers and community organization representatives, we noted the importance ascribed to “Netiquette”—digital social norms that govern digital communication undertaken with contacts [51]. Netiquette denoted survey requests as a type of spam. Research into “Netiquette” and the acceptability of receiving a survey request from a contact, may further clarify the effectiveness of WEB RDS for studying men who have sex with men and other populations Leverage saliency theory suggests that the wording of the request and which aspects of the study are emphasized in the request are crucial [52], and this effect could be even stronger among populations encountering a large number of surveys invitations and experiencing survey invitations as spam. An accessible way to study the impact of invitation phrasing by respondent recruiters would be to ask respondents to share their invitation texts and compare those to the (non-) response.

#### A Digitally but Not Geographically Connected Population

One major benefit of WEB RDS compared to offline RDS is access to a digitally connected population spread over a larger geographical area, such as a country [18]. This can, however, present challenges for population inference, as an important requirement is that the entire population under study is part of the same social network [33]. We found that community experts did not think men who have sex with men across the nation to be one network, but rather that networks are confined to a province or city. Recruitment homophily on the living environment (city or rural) was found to be modest. The qualitative findings are in line with those from WEB RDS studies in Sweden and Vietnam on geographical clustering [17,26]. A high degree of clustering implies that longer recruitment chains are needed for estimates to stabilize than previously thought [33]; this may be a bottleneck for nationwide WEB RDS of men who have sex with men in HICs, as reaching long chains has proven problematic [26,53,54]. Future studies should include a measure of location to monitor recruitment and determine if population inference over the desired geographical area is viable.

#### Men Who Have Sex With Men Who Were Not Open About Having Sex With Men

The fact that the population at increased risk of HIV and sexually transmitted infections (such as closeted men who have sex with men and men who have sex with men who have sex with anonymous partners) may not be reached through WEB RDS is especially relevant in the field of sexual health [55-58]. Community organization representatives did not expect these populations to be reached. Our WED RDS diagnostics did not show recruitment bias or differences in network size among men who have sex with men with occasional partners, and these men who have sex with men were represented in the final sample. However, as a proxy for men who have sex with men with only anonymous partners, it has its limitations. Offline RDS studies have been able to include men who have sex with men who were not out or open about having sex with men [54] and one offline RDS was able to include more men who have sex with men who did not identify as gay than a simultaneous time-location-(venue)-based sampling [59]. At least a portion of men who have sex with men who have anonymous sex contact with their sex partners through the internet, and this could facilitate WEB RDS [58]. Future WEB RDS among men who have sex with men aiming to include these populations may want to include variables on the level of outness, anonymity of sex partners, sharing contact information with other men who have sex with men, and how often they meet other men who have sex with men in person. An offline RDS or hybrid form could be explored to include those men who have sex with men who do not share contact information with other men who have sex with men.

#### Potential of Combining WEB RDS With Web-Based Convenience Sampling

The unique respondent-driven recruitment mechanism of WEB RDS likely led to some differences in sample composition compared to the EMIS-convenience sample. Our results show that men who have sex with men recruit those similar in age and urban environment, indicating that the composition of a WEB RDS sample (one that does not reach stabilized estimates) can be controlled through characteristics of the first respondents in the chain. Further studies are needed to determine how WEB RDS recruitment behavior impacts sample composition. Combining web-based convenience sampling with selective WEB RDS should be investigated further; when bias in web-based convenience sampling is expected, participants with underrepresented traits could be asked to recruit others through WEB RDS to mitigate this bias.

### Conclusions

To conclude, our study has shown that WEB RDS, even with a moderate incentive, is not a viable sampling strategy for obtaining national population estimates of sexual health traits among men who have sex with men in Belgium. We found that while a double incentive structure aided in stimulating participation, it was not sufficient to overcome challenges such as overresearch, low motivation for the topic, and netiquette. These challenges hindered the number of participants, number of waves, and therefore stabilization of the estimates. The study emphasizes the need for understanding men who have sex with men’s research motivation, exploring optimal incentive levels, addressing or overcoming overresearch, and considering alternative methods, such as offline RDS or hybrid approaches, to ensure the representation of diverse subpopulations. Finally, this study showcases the effective use of the modified MRC process evaluation as a tool for evaluating WEB RDS sampling processes.

## Appendix

Multimedia Appendix 1 Abbreviated STROBE-RDS (Strengthening the Reporting of Observational Studies in Epidemiology for Respondent-Driven Sampling) report for study design.

Multimedia Appendix 2 Qualitative data table.

Multimedia Appendix 3 Descriptions of WEB RDS specific indicators.

Multimedia Appendix 4 Calculation of target sample size.

Multimedia Appendix 5 All-points plot place of residence.

Multimedia Appendix 6 All-points plot occasional partners.

Multimedia Appendix 7 All-points plot education.

Multimedia Appendix 8 Seed mapping.

Multimedia Appendix 9 Sample comparison.

## Abbreviations

EMIS: European Men Who Have Sex with Men Internet Survey
HIC: high-income country
LGBTQI+: lesbian, gay, bisexual, transgender, queer/questioning, intersex, and others
MRC: Medical Resource Council
RDS: respondent-driven sampling
STROBE-RDS: Strengthening the Reporting of Observational Studies in Epidemiology for Respondent-Driven Sampling
WEB RDS: web-based respondent-driven sampling

## Data Availability

The datasets analyzed and the coding used during this study are available from the corresponding author on reasonable request.
