# Supplementary material for: Viability of Web-Based Respondent-Driven Sampling of Belgian Men Who Have Sex With Men: Process Evaluation
Source: J Med Internet Res. 2025 May 5;27:e60884. doi: 10.2196/60884 (PMC12089861; doi:10.2196/60884)
Supplement: Multimedia Appendix 1 [file jmir_v27i1e60884_app1.docx]

| MRC framework | Reporting criteria | Pilot | Main WEB RDS study |
| --- | --- | --- | --- |
| Context | Reason for RDS as the sampling method | MSM as a study population having no sampling frame | |
|  | Survey Topic | Sexual health | |
|  | Population;  Eligibility criteria | 18+; Living in Flanders or Brussels; Dutch, French, or English language; Self-identify as men or transwomen who have sex with men or transwomen; Sex with at least one man in the last 12 months; Able to provide informed consent. | |
| Implementation | Seed recruitment, selection, and number | Seeds were recruited through interest groups, researchers’ social networks and re-contacting participants of a previous study. Due to difficulty recruiting seeds and halting recruitment, we were obliged to use all seeds recruited without selection on criteria of good seeds. | |
|  |  | Number of invited seeds: 24 | Number of invited seeds: 38 |
|  |  | First seed invited: 04/30/2021 | First seed invited: 02/28/2022 |
|  |  | Last seed invited: 06/05/2021 | Last seed invited: 03/28/2022 |
| Mechanisms of impact | Number of invites | 4 | 6 |
|  | Recruitment options | Sharing a URL on a platform of choice, WhatsApp, or email and could be opened from the survey website. An email could also be sent from a system email-address. Survey was optimized for smartphones. | Sharing a URL on a platform of choice, WhatsApp, or email and could be opened from the survey website.  Survey was optimized for smartphones. |
|  |  | Provided standardized or personal invitation. | Personal invitation only. |
|  | Reminders | None. | Reminders consisted of messages sent as part of the remuneration email, with either: (A) a request to recruit if they had not done so, or (B) a request to remind their recruits to participate, in cases were the recruits had not done so. We included the specific links generated that had not been responded to. |
|  | Incentives | None. | Double incentive system, value ranging EUR 10–30 as an electronic gift card. |
