# Supplementary material for: Viability of Web-Based Respondent-Driven Sampling of Belgian Men Who Have Sex With Men: Process Evaluation
Source: J Med Internet Res. 2025 May 5;27:e60884. doi: 10.2196/60884 (PMC12089861; doi:10.2196/60884)
Supplement: Multimedia Appendix 2 [file jmir_v27i1e60884_app2.docx]

| **Type of respondent** | **Data collection** | **Practical information** |
| --- | --- | --- |
| **Community expert** | Interviews | 4 external community experts  Duration: +/- one hour |
| **Community stakeholder** | Focus group discussion | 4 FGD One moderator and 4-7 participants  Duration: +/- one hour |
| **Community stakeholder** | Consultations | 11 community stakeholders from 9 community organisations  Biannual meetings over a period of 4 years |
| **RDS expert** | Interviews | 1 Internal expert and 3 external experts.  Duration: +/- one hour |
| **RDS expert** | Consultations | Bimonthly consultations with 5 external RDS experts over a period of two years.  Annual consultations with 2 additional external RDS experts over a period of 4 years. |
| **Target population** | Pilot | 10 respondents |
| **Target population** | Interview | Recontacting all the seeds (16) of RDS-I;  7 respondents.  Duration: +/- 20 mins. |
