# Supplementary material for: Viability of Web-Based Respondent-Driven Sampling of Belgian Men Who Have Sex With Men: Process Evaluation
Source: J Med Internet Res. 2025 May 5;27:e60884. doi: 10.2196/60884 (PMC12089861; doi:10.2196/60884)
Supplement: Multimedia Appendix 3 [file jmir_v27i1e60884_app3.docx]

| **Nr.** | **Indicator** | **Description** |
| --- | --- | --- |
| S1 | Country | Many aspects of the country in which a WEB RDS is set can influence the mechanisms of impact, such as income level and available population specific resources |
| S2 | Population | In WEB RDS, the network structure of the population (one or multiple networks, clustering, bottlenecks) determines how much data needs to be gathered for population inference to be valid. |
| S3 | Topic | The topic can interact with the mechanisms of impact and performance indicators in various ways. |
| I1 | Seed recruitment | Seeds are participants recruited by the researcher, who initiate the sampling process by inviting peers. Seed recruitment refers to the ways in which researchers find and recruit these initial participants. |
| I2 | Seed selection | The rationale behind selecting specific participants from the seed pool (potential participants collected by the researcher) to become a seed. |
| I3 | Number of seeds | Number of participants recruited by the researcher and asked to invite peers. |
| M1 | Incentives | The stimuli provided for participation and/or for each individual recruited. |
| M2 | Recruitment options | Way in which participants can invite peers (e.g. physical coupon, invite link, system email). |
| M3 | Random referral | Estimators assume participants invite a random peer from their network. This can be enforced by asking participants to list all peers they know, and having software pick a random peer from the list to invite. However this procedure can inhibit recruitment. |
| M4 | Coupons | An invitation that a participant can send to their peers. Coupons use unique identifier codes to link recruiters with their recruited. Number of coupons differs between studies. |
| M5 | Reminders | Messages send by researcher or WEB RDS software to remind those invited to participate and recruit. |
| M6 | Unexpected pathways | Mechanisms impacting participation and recruitment, which were not identified before sampling started. |
| P1 | Total participants recruited | Number of participants recruited by their peers e.g. the final sample size minus the seeds. |
| P3 | Successfully recruiting participants | Number of participants who recruited at least one peer into the study. |
| P4 | Stabilized estimates without bottlenecks | A stabilized estimate without bottlenecks suggests the estimate is no longer dependent on the traits of the seeds. An estimate has stabilized if it no longer changes as more data is collected. A bottleneck occurs if traits remain highly chain dependent, with a chain referring to the sequence of participants where each participant recruits the next. |
| P5 | Duration of data collection | Duration of peer to peer recruitment and duration of adding new seeds to the sampling process. |
| P6 | Inclusion of subpopulations | If formative research suggests that the population may not form one network, bottlenecks hindering inclusion of subpopulations, or members of subpopulation not able to participate for another reason, sample composition should be investigated for percentage of participants from subpopulations of interest. |
