# Supplementary material for: Viability of Web-Based Respondent-Driven Sampling of Belgian Men Who Have Sex With Men: Process Evaluation
Source: J Med Internet Res. 2025 May 5;27:e60884. doi: 10.2196/60884 (PMC12089861; doi:10.2196/60884)
Supplement: Multimedia Appendix 4 [file jmir_v27i1e60884_app4.docx]

Calculation of the total number of participants recruited (P1) that is sufficient to compensate for RDS design effect of 3 with a standard error of 0.03, as most recently recommended for RDS [1, 2].

We estimate the percentage of MSM at risk of Sexually Transmitted Diseases (STDs) (having more than 1 partner, not always using condoms) at 25%, based on The European MSM Internet Survey (EMIS) study results for Belgium [3]. We calculated the minimum sample size using the formulae outlined in Salganik (2006) [2], where P_A_ is proportion, n = sample size, se is standard error, and ‘deff’ refers to the design effect:

$$n=deff\times{(P}_{A}(1-P_{A}))/\left( se\left( \hat{P}_{A} \right) \right)^{2}$$

**References**

1. Johnston LG, Chen Y-H, Silva-Santisteban A, Raymond HF. An Empirical Examination of Respondent Driven Sampling Design Effects Among HIV Risk Groups from Studies Conducted Around the World. AIDS and Behavior. 2013 2013/07/01;17(6):2202–10. doi: 10.1007/s10461-012-0394-8.

2. Salganik MJ. Variance estimation, design effects, and sample size calculations for respondent-driven sampling. Journal of Urban Health. 2006;83(Suppl 1):98–112. doi: 10.1007/s11524-006-9106-x.

3. Vanden Berge W DJ, Detandt S, Pezeril C, Sergeant, M BS. The European Men who have sex with men Internet Survey (EMIS) 2017. . Bruxelles, Belgique Sciensano; Observatoire du sida et des sexualités (ULB), 2021 Contract No.: D/2021/14.440/07.
