# Supplementary figures and images for: Viability of Web-Based Respondent-Driven Sampling of Belgian Men Who Have Sex With Men: Process Evaluation
Source: J Med Internet Res. 2025 May 5;27:e60884. doi: 10.2196/60884 (PMC12089861; doi:10.2196/60884)

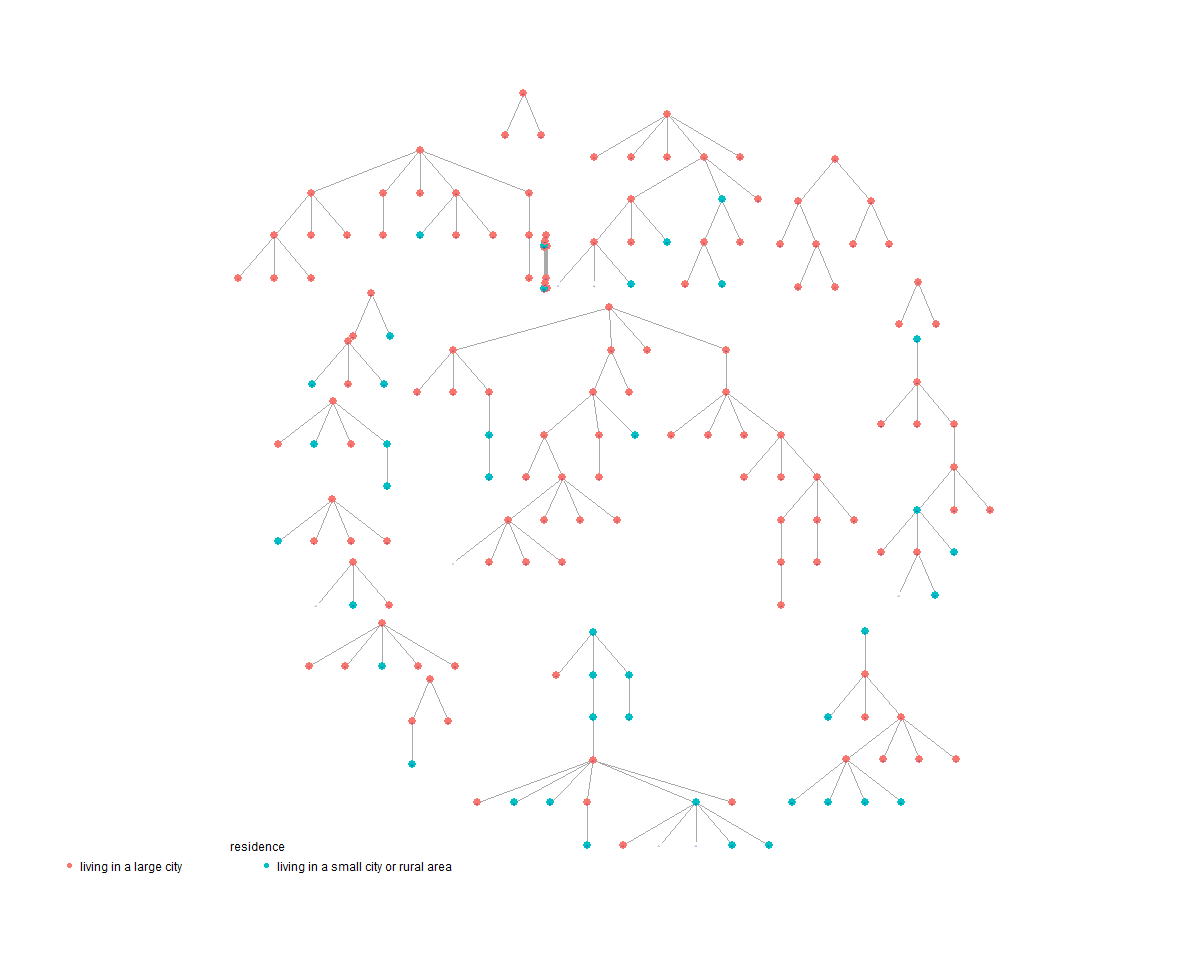

Supplement: Multimedia Appendix 5 [file jmir_v27i1e60884_app5.png]

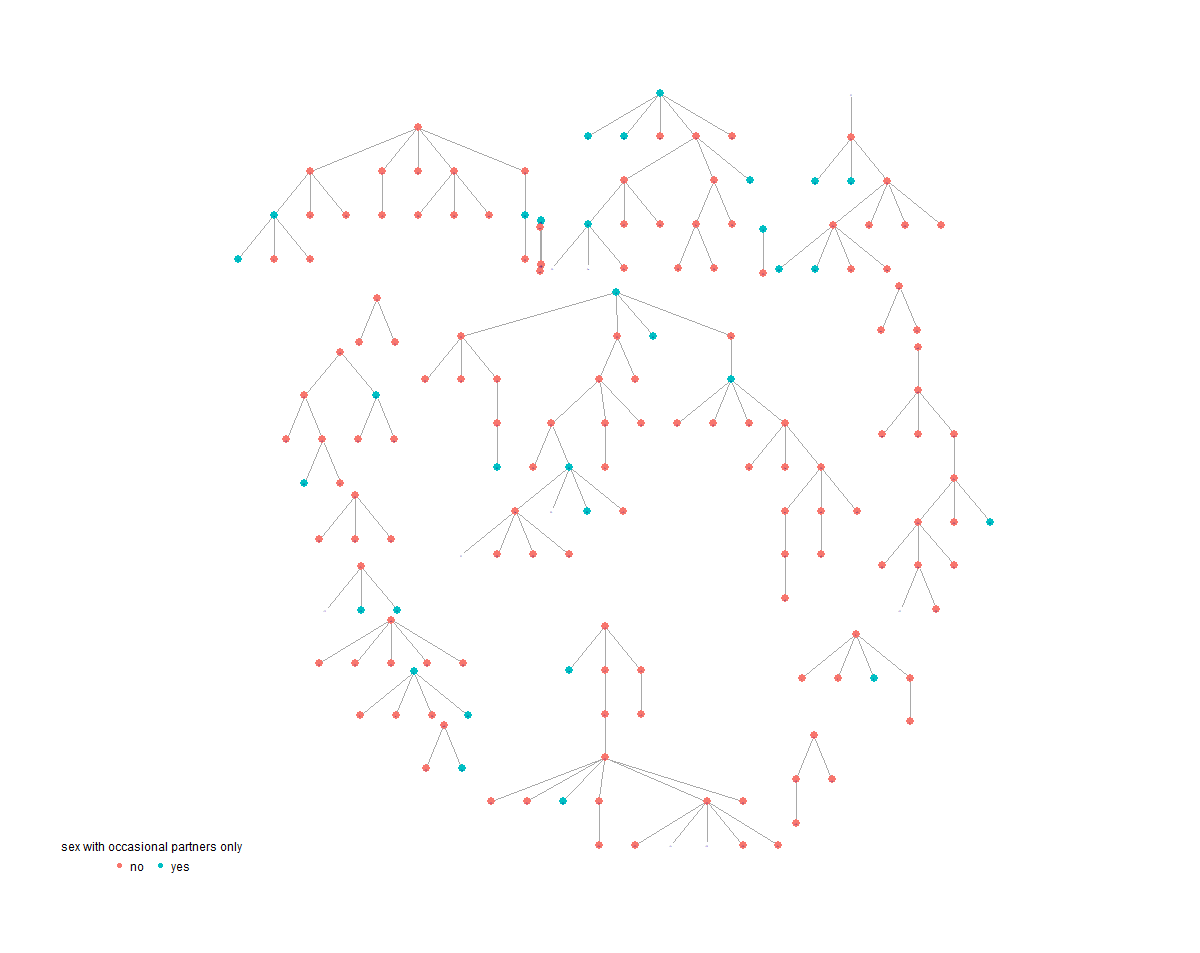

Supplement: Multimedia Appendix 6 [file jmir_v27i1e60884_app6.png]

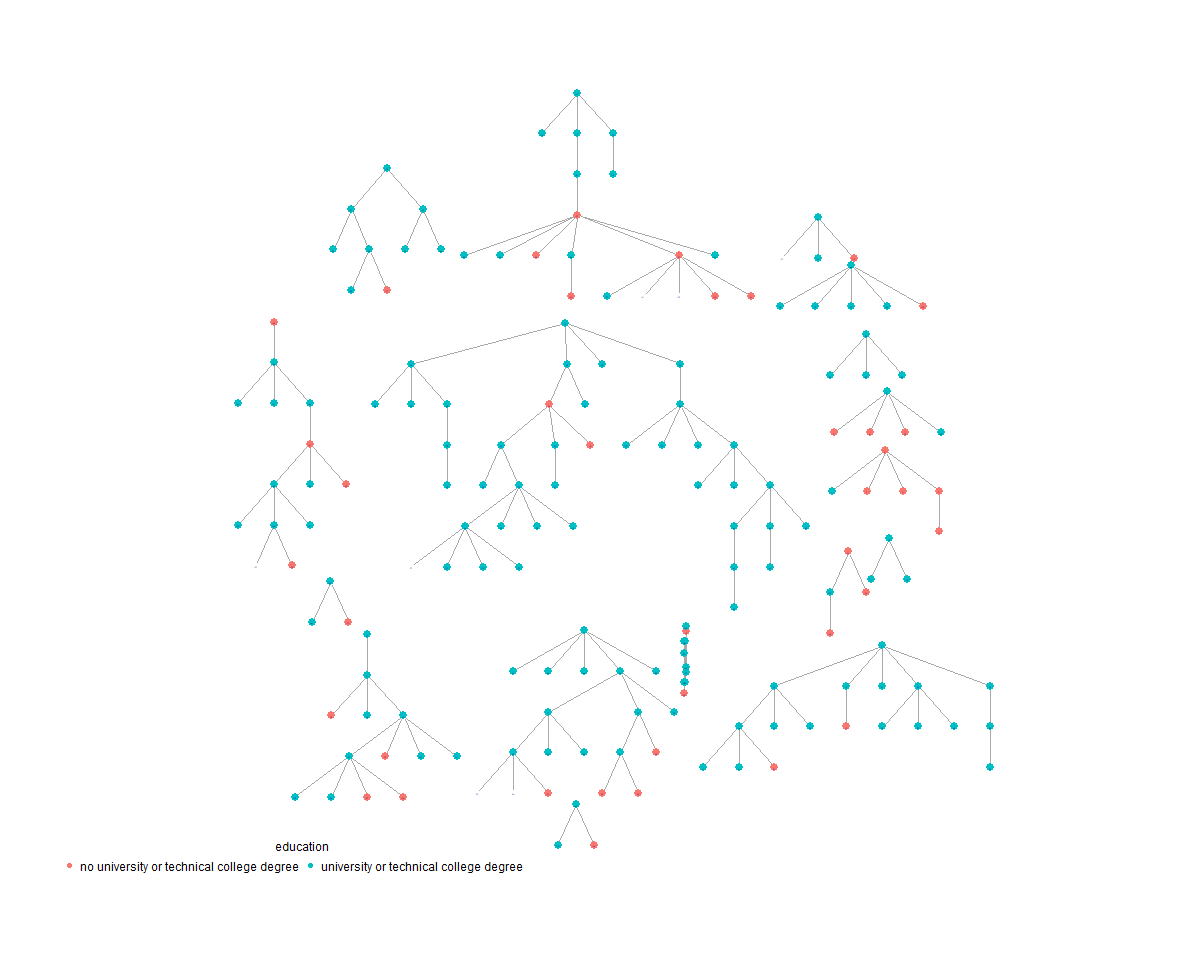

Supplement: Multimedia Appendix 7 [file jmir_v27i1e60884_app7.png]
