# Supplementary material for: Viability of Web-Based Respondent-Driven Sampling of Belgian Men Who Have Sex With Men: Process Evaluation
Source: J Med Internet Res. 2025 May 5;27:e60884. doi: 10.2196/60884 (PMC12089861; doi:10.2196/60884)
Supplement: Multimedia Appendix 8 [file jmir_v27i1e60884_app8.docx]

| Seed characteristic | | N |
| --- | --- | --- |
| Place of residence | Average to large city | 27 |
|  | Rural or small town, small city | 11 |
| Education | College or university degree | 30 |
|  | No College or University Degree | 8 |
| Age (years) | Thirty or over | 24 |
|  | Under Thirty (18+) | 14 |
| Place of Residence | Antwerp province | 8 |
|  | East-Flanders | 12 |
|  | Brussels capital region | 8 |
|  | Limburg | 3 |
|  | West-Flanders | 2 |
|  | Flemish-Brabant | 0 |
|  | Unknown | 5 |
| Only occasional partners | Yes | 9 |
|  | No | 25 |
|  | Unknown | 4 |
| Total |  | 38 |

Note: 4 seeds dropped-out before start of the sampling.
