# Supplementary material for: Viability of Web-Based Respondent-Driven Sampling of Belgian Men Who Have Sex With Men: Process Evaluation
Source: J Med Internet Res. 2025 May 5;27:e60884. doi: 10.2196/60884 (PMC12089861; doi:10.2196/60884)
Supplement: Multimedia Appendix 9 [file jmir_v27i1e60884_app9.docx]

| **Variables** | **WEB RDS sample** | | **EMIS BE** | |
| --- | --- | --- | --- | --- |
|  | **N** | **%** | **N** | **%** |
| **Age (years)** | **183** | **100.0** | **2242** | **100.0** |
| 18–29 | 77 | 42.1 | 492 | 21.9 |
| 30+ | 106 | 57.9 | 1750 | 78.1 |
| **Place of Residence** | **181** | **100.0** | **2226** | **100.0** |
| large/medium city (≥100000 persons) | 136 | 75.1 | 1280 | 57.5 |
| Village, countryside, small city/town (<100000 persons) | 45 | 24.9 | 946 | 42.5 |
| **Migration background** | **180** | **100.0** | **2242** | **100** |
| born in Belgium | 142 | 78.9 | 1790 | 79.8 |
| EU migrant | 21 | 11.7 | 273 | 12.2 |
| non-EU migrant | 17 | 9.4 | 179 | 8.0 |
| **Struggline with current income** | **181** | **100.0** | **2233** | **100.0** |
| not struggling | 174 | 96.1 | 2005 | 89.8 |
| struggling | 7 | 3.9 | 228 | 10.2 |
| **Attracted to men only** | **188** | **100.0** | **3012** | **100.0** |
| yes | 174 | 92.6 | 2584 | 85.7 |
| no | 14 | 7.4 | 428 | 14.3 |
| **Steady relationship(s)** | **184** | **100.0** | **2838** | **100.0** |
| yes | 110 | 59.8 | 1351 | 47.6 |
| no | 74 | 40.2 | 1487 | 52.4 |
| **HIV test ever** | **144** | **100.0** | **3016** | **100.0** |
| yes | 138 | 95.8 | 2207 | 73.2 |
| no | 15 | 4.2 | 809 | 26.8 |
| **STI test ever** | **184** | **100.0** | **2291** | **100.0** |
| yes | 168 | 91.3 | 2219 | 96.9 |
| no | 16 | 8.7 | 721 | 3.1 |
